# Prioritizing sexual and reproductive health in the face of competing health needs: where are we going?

**DOI:** 10.1186/s12978-021-01068-0

**Published:** 2021-01-15

**Authors:** Akaninyene Otu, Georges Danhoundo, Sanni Yaya

**Affiliations:** 1 Department of Infection and Travel Medicine, Leeds Teaching Hospitals NHS Trust, Leeds, UK; 2 Department of Internal Medicine, College of Medical Sciences, University of Calabar, Calabar, Cross River Nigeria; 3 Department of Sexual and Reproductive Health and Research, World Health Organization, Geneva, Switzerland; 4 School of International Development and Global Studies, University of Ottawa, Ottawa, ON Canada; 5 The George Institute for Global Health, Imperial College London, London, UK

A significant proportion of the world’s population does not have access to essential health services, incurs catastrophic health expenditure, and suffers impoverishment owing to out-of-pocket (OOP) expense. The World Health Organization (WHO) has identified “achieving Universal Health Coverage (UHC), with one billion more people benefitting from the coverage, as a strategic priority for achieving SDG 3: “Ensuring healthy lives and promoting well-being for all at all ages” [1]. Achieving UHC for sexual and reproductive health (SRH) is particularly challenging as UHC requires that countries make careful choices, and, depending on the context, many trade-offs may be called for. A combination of political commitment and well-defined/coherent financing and service provision approaches are essential [2].

In this editorial, we describe the evolution of SRH and highlight the significant gains of the global commitment to furthering the ideals of SRH. We identify existing gaps in the current SRH landscape and call attention to the urgent need to reprioritize SRH in the face of competing demands for scarce resources within the health sector. Finally, we provide pragmatic approaches to closing the identified gaps and upscaling SRH services.

## Reflecting on the journey thus far

Reproductive and sexual health were first defined at the 1994 International Conference for Population and Development (ICPD) as “a state of complete physical, mental and social well-being and not merely the absence of disease or infirmity, in all matters relating to the reproductive system and to its functions and processes” [3]. Significant progress has been made since the concept of reproductive and sexual health was defined and promoted at the ICPD in Cairo [4]. The emphasis on “all matters relating to the reproductive system and to its functions and processes” was further reaffirmed at the landmark 1995 United Nations (UN) Fourth World Conference on Women in Beijing. The conference in Beijing marked a significant turning point for the global agenda for reproductive health with the upscaling of actions aimed at promoting sexual health, reproductive rights, gender equality and women’s empowerment [5]. The World Summit of the UN General Assembly in 2005 reflected these ideals in three of the eight 2001 UN Millennium Development Goals with a focus on reducing child and maternal mortality and rolling back HIV/AIDS [6]. In 2015, the Sustainable Development Goals (SDGs) laid out a 15-year roadmap that included goals and targets relating to access to SRH services and comprehensive sexuality education (CSE) [7]. This was on the backdrop of a growing realization that SRH intersects with the three central dimensions of sustainable development namely social, economic and environmental. CSE refers to a curriculum-based process of teaching young people about the cognitive, emotional, physical and social aspects of sexuality [8].

## An unfinished agenda

The SDG target 3.1 aims at reducing global maternal mortality rate (MMR) to less than 70 per 100,000 live births by 2030. Despite the tremendous gains of the commitment from the international community to adressing SRH issues, there are still significant challenges. For example, the global MMR was reduced by 38% between 2000 and 2017 with an average global decline of 2.9% per annum. However, the maternal deaths in 2017 was 295,000 [uncertainty interval 80%: 279 000–340 000] with approximately 86% of the global mortality occurring in sub-Saharan Africa and South Asia [9]. This reflects wide disparities as women from low-income countries are estimated to have a 130 times higher risk of maternal mortality when compared with women living in a high-income country [10].

Referring to SDG target 3.7 “by 2030, ensure universal access to sexual and reproductive health-care services, including for family planning, information and education, and the integration of reproductive health into national strategies and programmes”, some progress has been made. For example, the proportion of women who have been provided with modern family planning methods has risen from 74 to 76 percent (SDG indicator 3.7.1) between 2000 and 2019 [11]. However, a significant gap persists as the proportion of women with unmet need for family planning has remained at 10 per cent since 2000 till present [11]. The number of women of reproductive age who desire to avoid pregnancy but do not use any contraceptive method has risen to 190 million from 156 million in 2000 [11]. This unmet need is particularly high among the adolescents, migrants, refugees, women in the postpartum period and urban slum dwellers [12]. Many countries have made efforts to integrate SRH services into national strategies and programmes, but a lot of work is still required to translate these policy measures into tangible gains.

The control of sexually transmitted infections (STIs) contributes towards the attainment of several SDGs namely SDG 3.2 (By 2030, end preventable deaths of newborns and children under 5 years); SDG 3.3 (By 2030, end the epidemics of AIDS, combat other communicable diseases); SDG 3.7 (By 2030, ensure universal access to sexual and reproductive health care); SDG 3.8 (By 2030, achieve universal health coverage) [12]. Although some inroads have been made into controlling STIs, the global burden of STIs remains unacceptably high as more than one million STIs are estimated to be acquired everyday worldwide. The WHO estimated that four curable STIs accounted for 376 million new infections in 2016—gonorrhea (87 million); chlamydia (127 million); syphilis (6.3 million); and trichomoniasis (156 million) [12]. Genital infection with herpes simplex virus (HSV) is estimated to affect over 500 million people and over 290 million women are estimated to have human papillomavirus (HPV) infection, the primary cause of cervical cancer [13]. Chronic hepatitis B is estimated to affect 240 million people globally and both HPV and hepatitis B are vaccine preventable infections. Some of these STIs like HPV and syphilis increase the risk of HIV transmission and many ultimately result in untoward consequences such as foetal losses, congenital infections and infertility.

About 1.8 billion, a sixth of the world’s population, is comprised of 10 to 24 year olds who have vast SRH needs at one of life's most complex stages [14]. Every day, an estimated 39 000 child marriages occur [15]. Annually, a projected 21 million girls aged 15–19 years in low-income countries become pregnant (most of which are unintended) [16]. Approximately 777,000 births occur to girls under 15 years of age in low-income countries [17]. Complications during pregnancy and childbirth account for the most deaths among 15–19-year-old girls globally; this is linked to the higher risks of eclampsia and puerperal infections among adolescent mothers. Among this age group, an estimated 5.6 million abortions occur annually with over 70% occurring in unsafe conditions [18]. The unmet need for family planning is greatest among adolescents who are also at high risk for contracting HIV and are more prone to sexual violence [19].

The WHO considers women’s cancer a major issue. Both breast and cervical cancer are responsible for more deaths in women than any other cancer in low-income countries. Early cancer detection and screening tests are still unavailable in most low-income settings. In 2018, an estimated 2.09 million deaths occurred from breast cancer and there were an estimated 570,000 new cases of cervical cancer in the same year with a resultant 311,000 deaths worldwide (90% of these occurred in low-middle income countries) [20]. Primary prevention of cervical cancer can be achieved with the aid of vaccination against HPV 16 and 18. The HPV vaccines have been introduced in 106 countries, but the global coverage is just 15% with a large population lacking access to this vaccine [21].

## Present day reality: conflicting demands for scarce resources

Unmet SRH needs pose a unique and monumental burden on society at large. Access to SRH services is vital to achieving SDGs goals. Across many countries, SRH services, which encompass a range of preventive, promotive and curative services across the life-course, are critical components of Primary Health Care (PHC). SRH services address broad and cross-cutting health needs which touch all aspects of health, at all ages, for all genders. SRH services also serve as entry points to the health system. For example, women's visits for SRH needs provide opportunities for screening for malaria, HIV or cervical cancer, and seeking care for gender-based violence (GBV) and sexual assault [22]. Despite the strong evidence for SRH services being a “best buy”, these services remain largely neglected and excluded from the priorities of many countries. SRH services have not always been on the agenda when global, and national-level discussions and decisions related to UHC are made, on financing health care, allocating resources and setting priorities [23]. A lack of structured approaches and rational allocation of resources coupled with a lack political commitment has militated against the achievement of many SRH goals. Discrimination on the basis of sex has led to many health disadvantages for women [24]. Taboos around sex and sexuality and the stigma attached to these issues have contributed to making SRH services a low priority in Health Benefits Packages (HBPs).

The challenges of SRH services have been exacerbated by the recent COVID-19 pandemic that has triggered the reallocation of resources and attention away from SRH with untoward consequences. The disproportionate impact on the health, wellbeing, and economic stability of women, girls, and vulnerable populations has become more obvious with the COVID-19 pandemic. SRH services are being deemed non-essential and providers reassigned to frontline roles to control COVID-19 [25]. For example, a recent survey conducted in Tunisia revealed that up to 50% of the SRH clinics in the country had been either reduced or suspended since COVID-19 emerged [26]. Global shortages of contraception are anticipated [27]. Social isolation measures aimed at slowing the spread of COVID-19 and reducing the risk to medical staff is also limiting the engagement of clients with SRH services.

The systemic racism, discrimination, and stigma that has characterized the COVID-19 pandemic is likely to further impact access to SRH care for women and vulnerable groups [28]. In the USA, elective surgeries are being deferred to limit the infectious exposure and conserve medical equipment [29, 30]. Funding and technical assistance by international organizations to the public sector for SRH is also taking a hit due to COVID-19. An upsurge of sexual GBV and unintended pregnancies have been recorded in Africa [31]. Lockdown measures and reduced access to contraceptives have contributed to the upsurge in unintended pregnancies in Africa. Limited access to HIV medications for women and pre-exposure prophylaxis (PrEP) for female sex workers has been reported [32]. In addition, interrupted access to education and skills acquisition for adolescent girls and young women (AGYW) remain a major challenge [33].

## Towards an inclusive future for SRH: suggested next steps

Inadequate integration of comprehensive SRH services in HBPs results in the burden of payment falling on service users that are already poor. Funding for SRH is mostly through OOP payments, with only a small proportion funded by domestic public funding. There is an urgent need to reprioritize SRH and make the services more resilient to shocks that emanate from disease outbreaks and competing health conditions. This cannot be achieved without securing political commitment from national stakeholders [34]. Promoting universal access to SRH services and incorporating SRH into existing national strategies and programmes will create integrated and comprehensive care packages. A substantial increase in SRH financing and development of a competent SRH workforce is required. Key actions are recommended:

- Ensuring global actors meet current funding commitments for SRH services;
- Increasing domestic public funding for SRH services;
- Improving efficiency and equity of existing resources;
- Strengthening integration of SRH in HBPs; and
- Ensuring an effective delivery of SRH services through accountability measures and community participation in the provision of these services.
